# Assessment of lymphatic filariasis prior to re-starting mass drug administration campaigns in coastal Kenya

**DOI:** 10.1186/s13071-017-2044-5

**Published:** 2017-02-22

**Authors:** Sammy M. Njenga, Henry M. Kanyi, Faith M. Mutungi, Collins Okoyo, Hadley S. Matendechero, Rachel L. Pullan, Katherine E. Halliday, Simon J. Brooker, C. Njeri Wamae, Joyce K. Onsongo, Kimberly Y. Won

**Affiliations:** 1 Eastern and Southern Africa Centre of International Parasite Control, Kenya Medical Research Institute, Nairobi, Kenya; 2 Neglected Tropical Diseases Unit, Ministry of Health, Nairobi, Kenya; 3 London School of Hygiene and Tropical Medicine, London, UK; 4 WHO Country Office, Nairobi, Kenya; 5 Department of Microbiology, School of Medicine, Mount Kenya University, Thika, Kenya; 6 Centers for Disease Control and Prevention, Atlanta, USA

**Keywords:** Lymphatic filariasis, *Wuchereria bancrofti*, Transmission assessment, Cross-sectional study, ICT test, Circulating filarial antigen, Microfilariae, Kenya

## Abstract

**Background:**

Lymphatic filariasis (LF) is a debilitating disease associated with extensive disfigurement and is one of a diverse group of diseases referred to as neglected tropical diseases (NTDs) which mainly occur among the poorest populations. In line with global recommendations to eliminate LF, Kenya launched its LF elimination programme in 2002 with the aim to implement annual mass drug administration (MDA) in order to interrupt LF transmission. However, the programme faced financial and administrative challenges over the years such that sustained annual MDA was not possible. Recently, there has been renewed interest to eliminate LF and the Kenyan Ministry of Health, through support from World Health Organization (WHO), restarted annual MDA in 2015. The objective of this study was to evaluate the current status of LF infection in the endemic coastal region of Kenya before MDA campaigns were restarted.

**Results:**

Ten sentinel sites in Kwale, Kilifi, Tana River, Lamu, and Taita-Taveta counties in coastal Kenya were selected for participation in a cross-sectional survey of LF infection prevalence. At least 300 individuals in each sentinel village were sampled through random house-to-house visits. During the day, the point-of-care immunochromatographic test (ICT) was used to detect the presence of *Wuchereria bancrofti* circulating filarial antigen in finger prick blood samples collected from residents of the selected sentinel villages. Those individuals who tested positive with the ICT test were requested to provide a night-time blood sample for microfilariae (MF) examination. The overall prevalence of filarial antigenaemia was 1.3% (95% CI: 0.9–1.8%). Ndau Island in Lamu County had the highest prevalence (6.3%; 95% CI: 4.1–9.7%), whereas sites in Kilifi and Kwale counties had prevalences < 1.7%. Mean microfilarial density was also higher in Ndau Island (234 MF/ml) compared to sentinel sites in Kwale and Kilifi counties (< 25 MF/ml). No LF infection was detected in Tana River and Taita-Taveta counties. Overall, more than 88% of the study participants reported to have used a bed net the previous night.

**Conclusions:**

Prevalence of LF infection is generally very low in coastal Kenya, but there remain areas that require further rounds of MDA if the disease is to be eliminated as a public health problem in line with the ongoing global elimination efforts. However, areas where there was no evidence of LF transmission should be considered for WHO-recommended transmission assessment surveys in view of stopping MDA.

## Background

In 2000, the World Health Organization (WHO) launched the Global Programme to Eliminate Lymphatic Filariasis (GPELF) in response to World Health Assembly resolution WHA50.29, which urged Member States to initiate activities to eliminate lymphatic filariasis (LF), a goal subsequently targeted for 2020 [1]. The GPELF has two principal aims: (i) to interrupt LF transmission, and (ii) to manage morbidity and prevent disability. To interrupt transmission of LF infection, the GPELF recommends annual community-wide mass drug administration (MDA) of antifilarial tablets to entire at-risk populations aged two years and above for 4–6 years at adequate levels of coverage. Modeling studies have estimated adequate treatment coverage to be at least 65% of total population in endemic areas [2, 3].

In Kenya, LF is confined to the coastal region where ecological factors are suitable for its transmission [4]. The Kenyan Ministry of Health (MoH) launched its LF elimination programme in 2002 when MDA was launched in the then Kilifi District. Unlike in many other African countries, onchocerciasis is not endemic in the LF endemic coastal Kenya. Therefore, the recommended antifilarial treatment for MDA is single-dose annual mass treatment with diethylcarbamazine citrate (DEC, 6 mg/kg) plus albendazole (400 mg). In 2003, the programme was scaled up to include Kwale and Malindi Districts. Another two rounds of MDA were conducted in these districts in March 2005 and December 2008 and a further round was conducted in December 2011, when MDA was extended to Tana River and Lamu counties. Such intermittent MDA is not consistent with GPELF recommendations to provide annual MDA for 4–6 years and its impact on transmission is unclear.

Monitoring and evaluation is recognized as an essential activity during implementation of any disease control programme. The current WHO guidelines for epidemiological monitoring of LF recommend selection of at least one sentinel site per 1 million people in the implementation unit (IU) [1]. The selected villages should have at least 500 persons so as to enable sample collection of at least 300 specimens. Testing for circulating filarial antigen (CFA) using immunochromatographic test (ICT) and parasitological detection of microfilariae (MF) in blood have been the gold standard tests for monitoring the impact of LF elimination programmes [1].

Kenya’s Ministry of Health NTD Unit successfully appealed to the World Health Organization Regional Office for Africa (WHO-AFRO) and other partners for support to re-establish the MDA programme starting in 2015. Subsequently, the WHO Country Office selected the Eastern and Southern Africa Centre of International Parasite Control (ESACIPAC), which is part of the Kenya Medical Research Institute (KEMRI), to conduct a comprehensive epidemiological assessment of LF infection before re-starting the MDA campaign in the coastal region of Kenya. The present paper reports results from this assessment and provides critical evidence that can be used for making decisions on MDA in addition to providing a basis for future monitoring of the LF elimination programme in coastal Kenya.

## Methods

### Study design and survey sites

A cross-sectional survey was conducted in October 2015 in ten LF sentinel sites (villages) located across the coastal region in Taita-Taveta, Kwale, Kilifi, Tana River and Lamu counties. Five of the sites were those that were previously selected by the LF elimination programme: Ndau Island (Lamu), Kipini (Tana River), Masindeni and Jaribuni (Kilifi), and Makwenyeni (Kwale). Five new sentinel sites were selected in Tana-River (Mikinduni), Kilifi (Kinarani), Kwale (Mirihini and Mwadimu), and Taita-Taveta (Kimorigo) to represent implementation units (sub-counties) that were established after initial MDA implementation. The five earlier sentinel sites were selected according to estimated risk of LF as estimated from a previously published report [5]. In the present study, health workers at the county level assisted in the selection of the 5 new sentinel sites. These new villages were purposively selected to participate in the survey based on the presence of cases of the disease and/or environmental factors indicating that LF transmission is likely to occur as given in the WHO-AFRO guidelines for mapping of lymphatic filariasis [6].

### Study population and sample size

The target population consisted of residents of the ten selected sentinel villages. The residents of villages in Taita-Taveta, Kwale, Kilifi and Tana River live in dispersed homesteads within their respective villages often located in the countryside. However, the residents of Ndau Island live in a relatively compact village with households being very close together. Typically, villages in the Kenyan coastal region have population of 600–900 persons [7]. Following WHO guidelines that at least 300 persons be tested in each sentinel site, the target sample population for the survey was 3,000 study participants. The sampling assumed that the average household size in coastal Kenya consists of 5 members per family and 3 individuals would agree to voluntarily participate in the survey. Thus, an estimated 100 households were to be visited in each village. Residents of the sentinel villages were recruited into the study if aged 2 years or more and not severely ill.

### Survey strategy

The LF survey was conducted using a house-to-house approach by four teams. Each team consisted of two laboratory technicians, two data collectors, a driver and a team leader. Additionally, the village chairman and a local volunteer in each selected village joined the survey team to assist with mobilization of community members. Individuals in each sentinel village were sampled through simple random house-to-house visits. Refusal to participate in the survey was encountered but the target sample was achieved in most sentinel sites.

A survey questionnaire was programmed onto mobile smartphones (Samsung Galaxy Trend S7560) and used to collect data from consenting participants (or parent/guardian in case of children). The data collected using the mobile smartphones included information on age, history of previous residence, use of deworming tablets, and long lasting insecticide-treated net (LLIN) ownership and use. Data on blood collection and results of the ICT test were also recorded onto the questionnaire. Additionally, the smartphones were used to collect global positioning system (GPS) coordinates of each study household.

### Laboratory procedures

#### Blood collection

The middle finger of consenting individuals was cleaned using a cotton ball soaked in 70% isopropyl alcohol. After drying, the tip of the finger was pricked using a sterile lancet and blood immediately collected using capillary tubes for ICT test (100 μl) and preparation of dry blood spots (DBS) on TropBio filter paper (60 μl). Serological tests will be performed later and described elsewhere. Any individual who tested positive for filarial antigens by ICT test, if consenting, was also tested for MF. Details of each laboratory procedure are given below.

#### Immunochromatographic test (ICT)

Prior to survey initiation, quality control (QC) of the ICT test kits (BinaxNow® Filariasis, Alere Inc., Orlando, USA) received for the survey was performed in KEMRI-ESACIPAC Regional NTD Reference Laboratory using well characterized serum samples. All the test kits assessed passed the QC analysis. In the field, 100 μl of the blood was used for the ICT test. After application of a whole blood sample to the ICT card, the results were read exactly at 10 min as recommended by the manufacturer. An additional 60 μl of finger prick blood samples were collected from participants and applied onto TropBio filter paper (TropBio Pty Ltd, Townsville, Queensland, Australia) for future serological studies.

#### Microfilariae detection

Individuals who tested positive by ICT test were invited for further testing for microfilariae in night time blood samples collected between 20:00 h and 24:00 h. The counting chamber method was used for examination and enumeration of *Wuchereria bancrofti* MF in the night blood specimens [8]. Briefly, 100 μl of blood was mixed with 900 μl of 3% acetic acid and the samples transported to KEMRI-ESACIPAC regional NTD reference laboratory in Nairobi where MF were examined and counted under a light microscope.

### Data management and analysis

Participants’ responses were captured electronically into Open Data Kit (www.opendatakit.org/), which included in-built data quality checks to prevent data entry errors.

Filarial infection was defined as a positive ICT result. Observed overall prevalence of filarial infection was calculated at sentinel site and county levels. 95% confidence intervals (CIs) were obtained by binomial logistic regression, taking into account clustering by households. Prevalence by sex and age group was calculated and 95% CIs determined using a Generalized Least Squares (GLS) random effects model that adjusts for household clustering. For purposes of this analysis, the following age groups were used: < 10, 10–17 and ≥ 18 year olds. The overall and village level proportion estimates of reported LLIN use were estimated and 95% CIs were determined using Generalized Linear Latent and Mixed Models (GLLAMM) adjusted for clustering by households. Overall, cross-county analysis of the impact of LLIN use on participant infection status was analysed, first using univariable analysis allowing for factors associated with filarial infection (i.e., age group and gender) and described as odds ratios (OR), using mixed effects logistic regression at both household and county levels. For multivariable analysis, adjusted OR (aOR) were obtained by mutually adjusting all minimum generated variables using multivariable mixed effects logistic regression at 95% CIs taking into account both household and county levels.

The mean coordinates of all households sampled in each village were used to obtain geographic locations of the sentinel sites that were mapped using Arc GIS Desktop version 10.2.2 software (Environmental Systems Research Institute, Inc., Redlands, CA). All statistical analyses were carried out using STATA version 14.0 (STATA Corporation, College Station, TX, US).

## Results

### Sentinel site surveillance

**Fig. 1:**
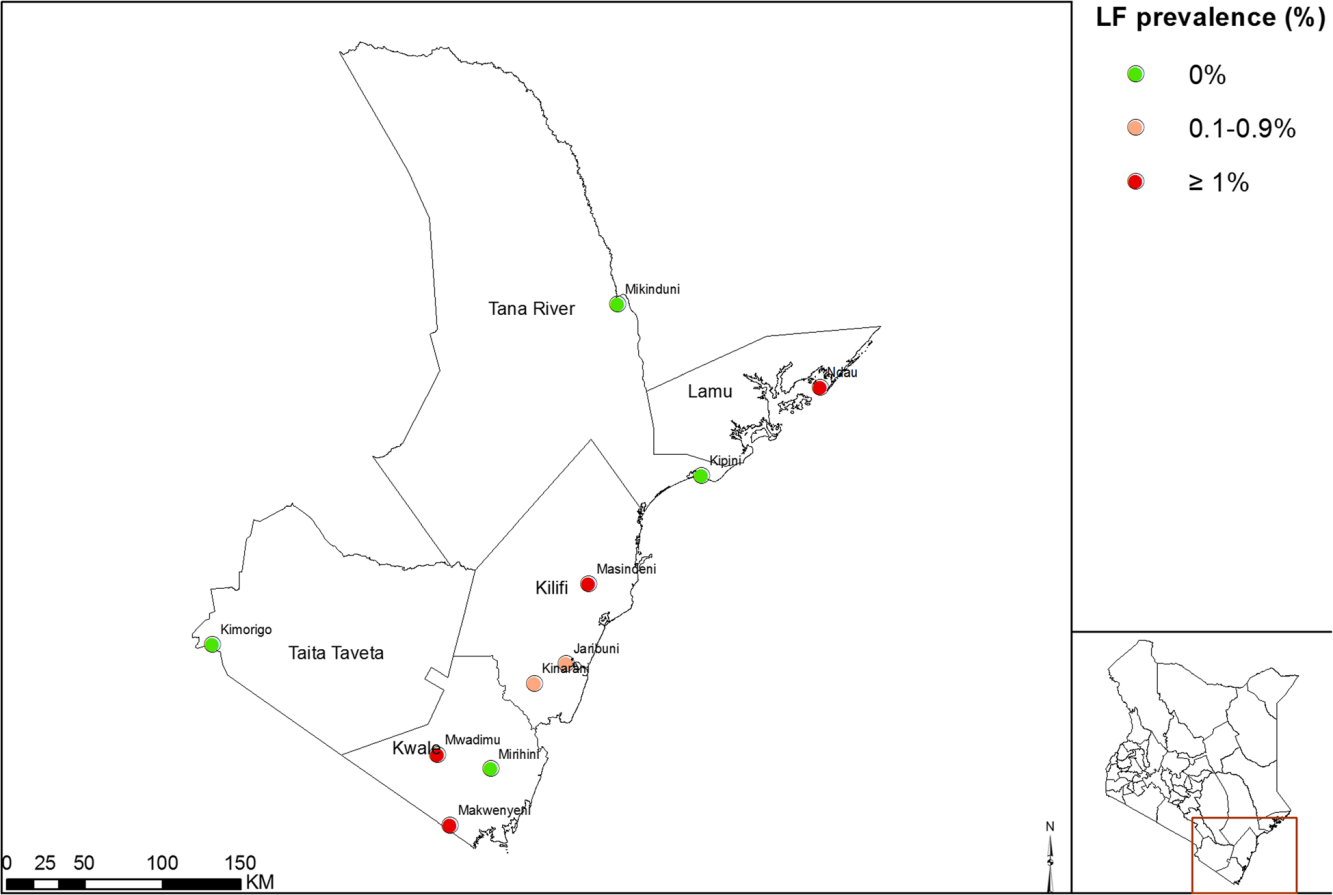
A map of the coastal region showing the location of the ten sentinel sites and lymphatic filariasis prevalence (%) levels by immunochromatographic test. The highest prevalence of lymphatic filariasis infection was detected in Ndau Island in Lamu County

Ten sentinel sites (villages) were surveyed between 8 and 18^th^ October 2015 in Kwale, Kilifi, Tana River, Lamu and Taita-Taveta counties in Coastal Kenya (Fig. 1). A total of 2,996 participants agreed to be registered for the survey, but 20 individuals (0.67%) either withdrew or did not provide a blood sample, hence final analysis was done for the remaining 2,976 participants. Samples for CFA testing using ICT test and dry blood spots (DBS) for serological assays were obtained and prepared for 2,976 participants and 2,972 participants, respectively. The reported age of individuals ranged from 2 to 100 years, with a median of 18 years (IQR = 31 years). Of the enrolled participants, 1,260 (42.3%) were male.

Table 1 provides the projected population of the five counties [9], demographic characteristics of the study participants, overall LF infection prevalence by ICT test in each county, and the adjusted odds ratios for the factors associated with the LF infection. Overall, 38 of 2,976 (1.3%; 95% CI: 0.9–1.8) individuals were found to be CFA positive using the ICT test. There was no significant difference in the prevalence of CFA positive individuals by sex (*P* = 0.148). Age-group classification was arbitrarily assigned for younger children (<10 year olds), older children (10–17 year olds), and adults (≥18 year olds). The odds of CFA among persons aged 18 years and above was significantly higher than those among younger persons (OR = 3.12; 95% CI: 1.16–8.43; *P* = 0.024). The overall prevalence of CFA positive persons in Kilifi and Kwale counties was 0.9% (95% CI: 0.4–1.8) and 1.1% (95% CI: 0.6–2.1), respectively, but there were villages where the prevalence was up to 1.7%. There was no evidence of LF infection in the sentinel sites in Tana River and Taita-Taveta counties.

**Table 1:** Demographic characteristics and filarial prevalence (%) by ICT test in 10 sentinel sites, coastal Kenya, October 2015

Demographic	2015 Population projections	Sentinel sites	*n* (%)	CFA prevalence (%) (95% CI)	Multivariable logistic	
					aOR (95% CI)^a^	*P*-value
County						
Kwale	792,698	3	877 (29.5)	1.1 (0.6–2.1)	–	–
Kilifi	1,307,185	3	911 (30.6)	0.9 (0.4–1.8)	–	–
Tana River	292,885	2	593 (19.9)	0	–	–
Lamu	123,842	1	320 (10.8)	6.3 (4.1–9.7)	–	–
Taita-Taveta	347,195	1	275 (9.2)	0	–	–
All counties	2,863,805	10	2,976	1.3 (0.9–1.8)	–	–
Sex						
Male	–	10	1,260 (42.3)	1.5 (0.9–2.4)	1.58 (0.85–2.95)	0.148
Female	–	10	1,716 (57.7)	1.1 (0.7–1.7)	Reference	
Age group						
< 10	–	10	865 (29.1)	0.7 (0.3–1.7)	Reference	
10–17	–	10	609 (20.5)	0.2 (0–1.2)	0.23 (0.03–2.05)	0.188
≥ 18	–	10	1,502 (50.5)	2.1 (1.5–2.9)	3.12 (1.16–8.43)	0.024*
LLIN use						
Yes	–	10	2,647 (88.9)	1.1 (0.8–1.6)	0.40 (0.19–0.86)	0.019*
No	–	10	329 (11.1)	2.7 (1.4–5.2)	Reference	

^a^Adjusted odds ratios (aOR) were obtained by mutually adjusting all minimum generated variables using multivariable mixed effects logistic regression at 95% CI taking into account households and county levels

*P < 0.05

Table 2 and Fig. 1 present the prevalence of CFA positive individuals by sentinel site. Ndau Island/village in Lamu County had the highest percentage of CFA positive persons, with 20 of 320 (6.3%; 95% CI: 4.1–9.7) individuals found to be antigen positive. Infection in Ndau Island was also observed in young children with 6 of the 20 (30%) CFA positive individuals being children aged 10 years and below.

**Table 2:** Surveyed households and sentinel site level circulating filarial antigen (CFA) prevalence (%), coastal Kenya, October 2015

County/Village	Households	No. CFA positive/No. examined	Prevalence (%) (95% CI)
Kwale County			
Makwenyeni	69	5/297	1.7 (0.7–3.9)
Mwadimu	67	5/290	1.7 (0.7–4.0)
Mirihini	52	0/290	0
Kilifi County			
Kinarani	94	1/307	0.3 (0–2.4)
Jaribuni	93	2/298	0.7 (0.2–2.6)
Masindeni	96	5/306	1.7 (0.7–3.9)
Tana River County			
Mikinduni	75	0/294	0
Kipini	83	0/299	0
Lamu County			
Ndau	105	20/320	6.3 (4.1–9.7)
Taita-Taveta County			
Kimorigo	94	0/275	0
All villages	828	38/2,976	1.3 (0.9–1.9)

Out of the 38 persons found to be positive for LF infection by ICT test, 33 (86.8%) provided a night-time blood sample for examination of MF. Assuming that all the individuals that were CFA negative by the ICT test were also negative for microfilaraemia, the prevalence of MF was highest in Ndau Island in Lamu County (1.9%; 95% CI: 0.9–4.1), but below 1% in three sentinel sites found to have CFA positive individuals in Kwale and Kilifi counties. The mean intensity of microfilaremia among MF positive persons in Ndau Island was also higher (234 MF/ml; 95% CI: 62–880) than in the other sentinel sites (Table 3).

**Table 3:** Sentinel site microfilariae prevalence (%) and mean intensity (MF/ml), coastal Kenya, October 2015

Village	No. CFA positive/No. examined	No. examined for MF^a^	No. MF positive	Mean intensity^b^ (MF/ml) (95% CI)	MF prevalence^c^ (95% CI)
Kwale County					
Makwenyeni	5/297	5	1	22 (3–156)	0.3 (0–2.4)
Mwadimu	5/290	4	1	10 (1–71)	0.3 (0–2.4)
Mirihini	0/290	0	0	0	0
Kilifi County					
Kinarani	1/307	0	0	0	0
Jaribuni	2/298	1	0	0	0
Masindeni	5/306	4	1	5 (1–35)	0.3 (0–2.4)
Tana River County					
Mikinduni	0/294	0	0	0	0
Kipini	0/299	0	0	0	0
Lamu County					
Ndau	20/320	19	6	234 (62–880)	1.9 (0.9–4.1)
Taita Taveta County					
Kimorigo	0/275	0	0	0	0
All villages	38/2,976	33	9	140 (39–502)	0.3 (0.2–0.6)

^a^Only CFA positive individuals were examined for MF by microscopy

^b^The mean intensity of MF was calculated among the CFA positive participants only

^c^All CFA negative individuals were assumed to be negative for MF and thus included in the calculation of MF prevalence

### Bed nets and deworming

Table 4 summarizes bed net ownership and usage among the 10 sentinel villages. Overall, 97.6% (95% CI: 96.6–98.5%) of the respondents reported owning at least one LLIN, with 88.8% (95% CI: 87.0–90.7%) reporting to have slept under a bed net the previous night. However, bed net usage was observed to be lower in Mwadimu village 73.3% (95% CI: 63.8–82.7) in Kwale County and Ndau Island 75.0% (95% CI, 67.9–82.1) in Lamu County. There was a significantly lower risk of LF infection among participants who reported bed net use compared to those who didn’t use a bed net (Table 1, OR = 0.40; 95% CI: 0.19–0.86; *P* = 0.019).

**Table 4:** Bed net ownership and usage by sentinel village, coastal Kenya, October 2015

Village	Proportion possessing at least one LLIN % (95% CI)	LLIN usage, previous night % (95% CI)
Makwenyeni	99.7 (99.0–100)	89.2 (84.0–94.4)
Mwadimu	95.1 (90.8–99.3)	73.3 (63.8–82.7)
Mirihini	91.5 (84.2–98.7)	89.5 (82.0–96.9)
Kinarani	97.4 (91.2–99.6)	89.6 (83.9–95.4)
Jaribuni	99.5 (98.6–100)	92.7 (88.1–97.3)
Masindeni	98.4 (93.0–99.1)	88.1 (82.8–93.5)
Mikinduni	99.0 (95.8–100)	93.6 (89.5–97.7)
Kipini	100 (98.6–100)	99.5 (98.5–100)
Ndau	98.7 (96.7–100)	75.0 (67.9–82.1)
Kimorigo	96.7 (94.2–99.3)	96.7 (94.4–99.0)
All villages	97.6 (96.6–98.5)	88.8 (87.0–90.7)

Of 2,950 responses about deworming, 1,184 individuals (40%) reported receiving deworming drugs during the last six months prior to the study with 68.6 and 21.0% receiving the treatment at school and home, respectively.

## Discussion

The results of the current survey suggest that transmission of LF infection in Tana River and Taita-Taveta counties may be absent and could be used to request WHO-AFRO to support the Kenyan LF programme to conduct transmission assessment surveys in these counties. Kenya’s LF elimination programme was launched in 2002, but has however, seen inconsistent treatment delivery coupled with challenges that resulted in MDA campaigns not being conducted every year as recommended by the GPELF (Table 5). A renewed commitment to re-start the LF elimination programme in Kenya attracted support from the WHO-AFRO Regional Office and other partners and an MDA campaign was conducted in October 2015. This study was undertaken to provide the status of LF infection in the Kenyan coastal region, which is required in order to inform decisions on MDA campaigns. Overall, ICT positivity in most sentinel sites ranged between 0 and 1.7%. However, the LF infection data in sentinel sites in Lamu, Kilifi and Kwale counties indicate that transmission is still ongoing in these counties, thus justifying additional rounds of MDA in the three counties. These data, therefore, could allow the programme to focus the currently available resources in areas that have empirical evidence of LF infection.

**Table 5:** MDA implementation in Coastal Kenya showing overall treatment coverage (%), 2002–2015

County	2002	2003	2005	2008	2011	2015
Kilifi	MDA	MDA	MDA	MDA	MDA	MDA
(Malindi)		MDA	MDA	MDA	MDA	MDA
Kwale		MDA	MDA	MDA	MDA	MDA
Tana River					MDA	MDA
Lamu					MDA	MDA
Taita-Taveta						
Programme (drug) coverage	81.2	79.5	72.3	62.7	58.3	54.3

The original IUs have been revised due to several changes in administrative structures. Malindi is currently a sub-county in Kilifi County. Source: WHO preventive chemotherapy database (WHO/PCT databank) http://www.who.int/neglected_diseases/preventive_chemotherapy/lf/en/ Accessed 06/11/2016

Ndau Island in Lamu County had a relatively higher infection rate (6.3%) compared to the sentinel sites on the mainland. The microfilarial density among MF positive persons was also relatively higher in Ndau Island compared to the other sentinel sites. Additionally, about 30% of LF infections on this island was detected in children aged ten years and below. Therefore, Ndau Island appears to be a hotspot of LF transmission and could be an indication of a similar situation in the other neighbouring islands. A previous study conducted in Ndau Island four years after a pilot MDA campaign found MF prevalence to be 13.7% [10]. A survey conducted by our team in 2011, prior to the first MDA in Lamu County under the LF elimination programme, found an MF prevalence of 11.6% (MoH, unpublished). The results of the current study, however, demonstrate that the MDA campaign conducted in 2011 may be associated with a reduced prevalence of LF infection in the Island. Nonetheless, further epidemiological studies in Ndau Island should be considered to identify factors responsible for continued transmission of LF infection. A study in Leogane, Haiti examined factors that could contribute to continued transmission of LF infection and found that MDA non-compliance was significantly associated with infection [11].

The current study found that most households possessed at least one bed net and the majority of people interviewed reported that they used the nets regularly. The high bed net possession was corroborated by observation of many new bed nets (some still unopened) during the current study because the national malaria control programme had conducted a mass LLIN distribution a few weeks prior to the LF survey. Vector control is increasingly being recognized as a possible complementary strategy for LF elimination [12–14]. A previous study found that vector control in Africa had increased significantly since 2005, with a three-fold increase in LLIN ownership and IRS coverage [15]. A few countries where there has been high LLIN coverage have reported the possibility of LF elimination in the absence of a MDA programme. For example, the Gambia has historical evidence of LF transmission [16–18], a long history of large scale bed net distribution [19, 20], and recent reports suggest that LF is no longer a public health problem in the country [21]. The current study observed significantly lower LF prevalence and risk of infection among individuals who reported bed net use thus suggesting that LLINs may have played a complementary role in reducing LF infection in the endemic Kenyan coastal region.

A study on the impact of permethrin-impregnated bed nets on LF vector mosquitoes in villages in Kwale County reported that LF is transmitted by both culicine and anopheline mosquitoes. Of the LF vector species collected before implementation of the intervention, 33.6% were members of *An. gambiae* complex [with more than 98% being *An. gambiae* (*sensu stricto*)], 30% were *An. funestus*, and 36.4% were *Culex quinquefasciatus* [22]. A malaria entomologic study reported that the primary vectors of malaria along the coast of Kenya include *An. funestus* and *An. gambiae* complex: *An. gambiae* (*s.s.*), *An. arabiaensis*, and *An. merus* [23]. The WHO promotes integrated vector management (IVM) to improve the cost effectiveness of vector-control operations, and to strengthen the capacity of programmes, partnerships and intersectoral collaboration in their efforts to control, eliminate or eradicate vector-borne diseases [24]. In areas with overlapping geographical distribution of LF and malaria, particularly where both infections are transmitted by the same species of mosquito vectors, the IVM approach is recommended as useful and appropriate for jointly managing control activities for the two diseases [25]. Although pyrethroid resistance has become widespread among anopheline and culicine mosquitoes [26–28], the sustained use of insecticide-treated bed nets has been associated with significant decrease in number of culicine mosquitoes in houses [29], which should therefore contribute to a reduction in LF transmission.

According to the 2010–2020 strategic plan of the GPELF, the strategic aim is to provide access to MDA and other measures to interrupt transmission in all endemic areas [30]. The current study provided further evidence that LLINs against malaria can indeed have complementary impact against LF and thus significantly contribute towards the goal to interrupt transmission of infection. This finding could be used to strengthen the call to adopt IVM approach which requires coordinated control of both malaria and lymphatic filariasis so that the two programmes could benefit from each programme’s activities, thus enhancing their overall impact on public health [24, 25]. Therefore, the Kenyan LF and malaria programmes should consider jointly undertaking mosquito vector control in the coastal region so as to enhance their overall impact on public health. This way, any residual LF transmission is likely to be completely eliminated.

Albendazole is a broad spectrum anthelmintic and is also used to treat LF infection, although the evidence on its efficacy when used alone is conflicting; studies in India demonstrated significant effects on both microfilariae and antigenaemia [31], but a study in Ghana reported minimal efficacy [32]. The current study found substantial use of deworming drugs, which could be due to the ongoing national school-based deworming programme that provides annual albendazole for the treatment of soil-transmitted helminths [33]. A recent study conducted in an informal settlement area in Nairobi revealed that there are many NGOs and religious organizations that also provide albendazole to school-age children in Kenya [34]. Nonetheless, the results of the current study are similar to those from previous work in a historically high LF endemic area in Malindi sub-County in Kilifi County, which reported sustained reduction in LF infection despite missing MDA rounds [7]. Taken together, the data suggest that LLIN use and deworming may have contributed to reduce LF infection despite the irregular implementation of MDA.

A number of tests are currently available for diagnosis of *W. bancrofti* infection but thick blood smear microscopy for detection of MF and ICT for testing for CFA were chosen for monitoring and evaluation of LF elimination programmes [1]. Previous evaluation of the ICT test in the coastal Kenya setting, before start of MDA campaigns, found the diagnostic tool to be 100% sensitive and specific for LF [35]. However, a study in Cameroon has reported loss of sensitivity of ICT test in low prevalence settings and raised concern regarding the use of this tool for monitoring and evaluation of LF elimination programmes [36]. Additionally, results of studies carried out in Central Africa have shown cross-reactivity of ICT test with *Loa loa* and *Onchocerca ochengi* infections and raised some doubts to the reliability of LF mapping data particularly in areas of *L. loa* co-endemicity [37, 38]. Therefore, the use of ICT test as the gold standard diagnostic tool in this study may be considered as a limitation that may significantly impact on the conclusions. Nonetheless, there are studies suggesting that antifilarial antibody testing could provide a more sensitive and specific measure of exposure to *W. bancrofti* in carefully selected populations in endemic areas and thus, may also be valuable as a tool for monitoring and evaluation of LF elimination programmes [39, 40]. Therefore, it might be useful to conduct operational research using strategies that complement CFA testing with the sensitive and specific antibody detection diagnostic assays to provide further information on current LF transmission in these counties.

## Conclusion

The current study suggests that LF transmission may be absent in Taita-Taveta and Tana River counties in coastal Kenya and therefore transmission assessment surveys (TAS) should be considered with a view to stopping MDA. By contrast, evidence for ongoing transmission in Kwale, Kilifi and Lamu counties indicates the need for further MDA rounds in these counties.

## Abbreviations

CI: Confidence interval
ESACIPAC: Eastern and Southern Africa Centre of International Parasite Control
GLLAMM: Generalized linear latent and mixed models
GPELF: Global programme to eliminate lymphatic filariasis
ICT: Immunochromatographic test
IVM: Integrated vector management
KEMRI: Kenya Medical Research Institute
LF: Lymphatic filariasis
LLIN: Long lasting insecticide-treated net
MDA: Mass drug administration
MF: Microfilariae
MoH: Ministry of Health
NTDs: Neglected tropical diseases
QC: Quality control
WHO: World Health Organization
